# Ecoregional Dominance in Spatial Distribution of Avian Influenza (H5N1) Outbreaks

**DOI:** 10.3201/eid1308.070329

**Published:** 2007-08

**Authors:** Raja Sengupta, Lauren Rosenshein, Mark Gilbert, Claire Weiller

**Affiliations:** * McGill University, Montreal, Quebec, Canada

**Keywords:** Geographic information system, Anatidae, influenza, H5N1, ecoregions, letter

## To the Editor

Recent articles in Emerging Infectious Diseases (*1*,*2*) and elsewhere (*3*,*4*) have highlighted the role of Anatidae migration in dispersal of the H5N1 subtype of highly pathogenic avian influenza (HPAI) virus. Although these articles point out that identifying the geographic origin of migrating waterfowl is needed to understand and predict pathogen dispersal, study analyses have been limited to pathways with nominal reference to climatic and vegetation patterns that control spatiotemporal patterns of this migration.

We propose that a better understanding of the threat of future spread can be obtained by identifying specific climatic and vegetation zones that are important in the life cycle of Anatidae, and which account for a disproportionately large number of HPAI outbreaks. The concept of ecoregions (*5*,*6*), i.e., distinct assemblages of natural communities determined by climate, geology, and evolution, is a useful zonal classification for evaluating HPAI outbreaks. A World Wildlife Fund classification delineating 825 terrestrial ecoregions (*7*), combined with a Google Earth map of 3,133 avian influenza outbreaks from November 24, 2003, to November 21, 2006 (*8*), provided the basis for this analysis. All files were converted to shapefiles (Environmental Systems Research Institute, Redlands, CA, USA), and overlay analysis was performed by using ArcGIS software (Environmental Systems Research Institute).

The Appendix Figure shows a chloropleth map (display of quantitative or qualitative information about subentities in terms of symbols or colors) of ecoregions with numbers of avian influenza cases (each spatially and temporally isolated set of individual events, regardless of number of deaths, is recorded as a case). Panels A, B, and C of this figure show enlargements of specific ecoregions with large numbers of known cases in regions of Eurasia, Southeast Asia, and Africa, respectively. Twenty-five ecoregions, representing 8.8% of the terrestrial surface area, accounted for 2,407 (76.8%) cases. A total of 132 of 825 ecoregional classifications had >1 recorded case of an avian influenza outbreak, but most (83) had <10 cases each.

Regionally, Southeast Asia has 12 ecoregions that collectively account for 1,651 cases (Appendix Figure, panel B) that have occurred consistently, albeit cyclically, since 2003. Among these ecoregions, the freshwater wetlands of the Chao Phraya, Tonle Sap, and Red Rivers are known migratory waterfowl wintering habitats in which 719 cases were located. Recent phylogenetic evidence suggests that this area is a local hotspot for an endemic strain of avian influenza (H5N1) that demonstrates bidirectional dispersal among localities within the region (*9*).

In the Eurasian region (Appendix Figure, panel A), 12 ecoregions accounted for 712 cases. The easternmost ecoregions, the Kazakh forest steppe (location of Lake Chany, an Anatidae habitat and breeding area) and the Kazakh Steppe, accounted for 132 cases, with the first case recorded on July 18, 2005. Subsequent major outbreaks in this region occurred in July–August 2005 and December 2005–January 2006. Regions around the Black Sea, including Euxine-Colchic broadleaf forests (deltas of the Kizil and Yesil Rivers), westernmost Pontic steppe (Lake Sivash), and Balkan mixed forests (deltas of the Danube, Olt, and Siret Rivers) have been loci for outbreaks in the central Eurasian region since October 1, 2005. Additional outbreaks have occurred since October 21, 2005, in the Eastern Anatolia montane steppe and deciduous forests (location of Lakes Van and Urmia, and Karakaya and Keban Baraji Reservoirs). Mixed and broadleaf forests of central and western Europe account for the remaining European cases since October 19, 2005. Anatidae habitats in this area include freshwater wetlands formed by the Danube, Rhine, Rhone, and Saone Rivers, and the Baltic basin.

Two African ecoregions, the Nile Delta–flooded savanna (Appendix Figure, panel A) and the West Sudanian savanna (Appendix Figure, panel C), including part of the Lake Chad ecosystem, i.e., the Kano River and the Tiga Reservoir, accounted for 79 cases. The initial Sudanian savanna case was identified on January 10, 2006, and the initial Nile Delta case was identified on February 17, 2006.

Our results may be skewed by several confounding factors, e.g., low national surveillance capabilities resulting in unreported cases and effects of the poultry trade. Nonetheless, the findings have implications for global monitoring of avian influenza (H5N1) outbreaks. Although migratory pathways and the poultry trade should continue to be scrutinized, monitoring efforts should focus on wintering and breeding habitats of migrating waterfowl, especially wetlands located within ecoregions with a disproportionately large number of avian influenza outbreaks. These hotspots are also likely to give rise to endemic local strains with regional dispersal characteristics (*9*).

## Supplementary Material

Appendix Figure Twenty-five ecoregions with large numbers of avian influenza cases (November 2003-November 2006). A) Eurasia; B) Southeast Asia; C) Africa. Yellow regions are composed of aggregated dots representing individual cases.
