# Supplementary figures and images for: Ecoregional Dominance in Spatial Distribution of Avian Influenza (H5N1) Outbreaks
Source: Emerg Infect Dis. 2007 Aug;13(8):1269–71. doi: 10.3201/eid1308.070329 (PMC2828375; doi:10.3201/eid1308.070329)

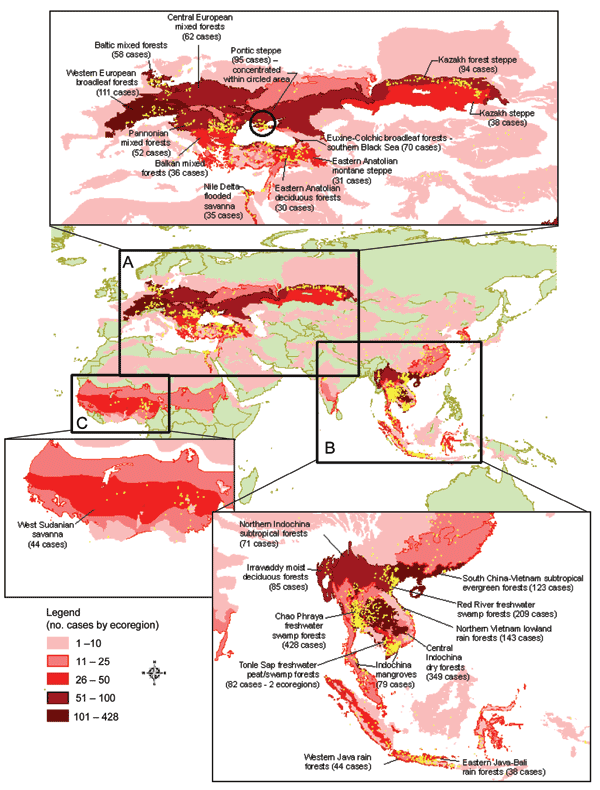

Supplement: Appendix Figure — Twenty-five ecoregions with large numbers of avian influenza cases (November 2003-November 2006). A) Eurasia; B) Southeast Asia; C) Africa. Yellow regions are composed of aggregated dots representing individual cases. [file 07-0329_appF-s1.gif]
