# Supplementary material for: Ecoregional Dominance in Spatial Distribution of Avian Influenza (H5N1) Outbreaks
Source: Emerg Infect Dis. 2007 Aug;13(8):1269–71. doi: 10.3201/eid1308.070329 (PMC2828375; doi:10.3201/eid1308.070329)
Supplement: Related texts

# Reply: In Response

**Authors:** Marius Gilbert, Xiangming Xiao, Jan Slingenbergh

**Affiliations:** * Free University of Brussels, Brussels, Belgium; † University of New Hampshire, Durham, New Hampshire, USA; ‡ Food and Agriculture Organization of the United Nations, Rome, Italy

Sengupta et al. (*1*) discuss the role of ecoregions in the distribution of HPAI (H5N1) outbreaks. Although the concept of ecoregions is undoubtedly useful in global biogeography, we do not understand the point they are trying to make. In our article (*2*), which is cited in their letter, we undertook a descriptive study to determine whether spread of HPAI (H5N1) virus was consistent in time with ecologic drivers of bird migration and in space with distribution of major migratory flyways of Anatidae. It is obvious that the distribution pattern of Anatidae is dependent on ecologic variables, and some of these variables are summarized by the ecoregion concept.

However, apart from a strictly descriptive point of view, we do not see how the ecoregion concept applies to describe patterns in HPAI (H5N1) spread and distribution. Sengupta et al. list ecoregions where reports of HPAI (H5N1) were concentrated. However, what do they infer from this? They observe regions with many reports of HPAI (H5N1) and conclude that these ecoregions are at risk. We find this reasoning completely circular, and any geographic zonation would provide the same observation. They may mean that ecoregions define boundaries within which secondary spread of HPAI is more likely than across ecoregions. However, this hypothesis would need to be more clearly demonstrated and quantified before the ecoregion concept can be used for global monitoring of HPAI (H5N1) outbreaks.

## References

[1] Sengupta R, Rosenshein L, Gilbert M, Weiller C. Ecoregional dominance in spatial distribution of avian influenza (H5N1) outbreaks. Emerg Infect Dis. 2007;13:1269–70.1795311510.3201/eid1308.070329PMC2828375

[2] Gilbert M, Xiao X, Domenech J, Lubroth J, Martin V, Slingenbergh J. Anatidae migration in the western Palearctic and spread of highly pathogenic avian influenza H5N1 virus. Emerg Infect Dis. 2006;12:1650–6.1728361310.3201/eid1211.060223PMC3372333

# Reply: In Response

**Authors:** Elsa Jourdain, Michel Gauthier-Clerc, Philippe Sabatier

**Affiliations:** * Station Biologique de la Tour du Valat, Arles, France; † Ecole Nationale Vétérinaire de Lyon, Marcy l’Etoile, France

Ecoregions are large units of land that reflect the distribution of species and communities and are used to define priority areas for biodiversity conservation. In their letter (*1*), Sengupta et al. suggest that ecoregions could help explain the spatial distribution of observed outbreaks of HPAI (H5N1). Since most outbreaks they observed occurred in ecoregions with waterfowl habitats, they concluded that monitoring efforts should focus on these areas.

Habitat mapping is useful for predicting the spread of wildlife-associated diseases. However, because ecoregions are large areas where agriculture and other human activities abound, many confounding variables exist. To assume that ecoregions with HPAI (H5N1) outbreaks are chiefly characterized by the natural biotopes is too simplistic.

In addition, the authors assume that wild waterfowl are the main factors in the dispersal of HPAI (H5N1). Although wild birds are known to be involved in the dispersal of low pathogenic strains of avian influenza viruses (*2*,*3*), the precise role of these birds in the dispersal of HPAI (H5N1) viruses remains controversial. It is now clear that the expansion of HPAI (H5N1) in Asia and to Africa was primarily associated with the poultry trade (*4*), which is not represented on a map of ecoregions. Also noteworthy is that HPAI (H5N1) did not persist in wild birds after the spring of 2006, and no case has been detected in the wetlands of America despite the migration of wild ducks and waders from Siberia (their main breeding ground) to Asia, Europe, and North America.

We believe that human activity is the primary vehicle for the spread of HPAI (H5N1) virus. Rather than the monitoring of waterfowl habitats, increased surveillance of the legal and illegal trade of birds and bird products is more likely to help in predicting the spread of this virus.

## References

[1] Sengupta R, Rosenschein L, Gilbert M, Weiller C. Ecoregional dominance in spatial distribution of avian influenza (H5N1) outbreaks. Emerg Infect Dis. 2007;13:1269–70.1795311510.3201/eid1308.070329PMC2828375

[2] Jourdain E, Gauthier-Clerc M, Bicout DJ, Sabatier P. Bird migration routes and risk for pathogen dispersion into western Mediterranean wetlands. Emerg Infect Dis. 2007;13:365–72. 10.3201/eid1303.06030117552088PMC2725901

[3] Olsen B, Munster VJ, Wallensten A, Waldenstro J, Osterhaus A, Fouchier R. Global patterns of influenza A virus in wild birds. Science. 2006;312:384–8. 10.1126/science.112243816627734

[4] Gauthier-Clerc M, Lebarbenchon C, Thomas F. Recent expansion of highly pathogenic avian influenza H5N1: a critical review. Ibis. 2007;149:202–14. 10.1111/j.1474-919X.2007.00699.x
